# Modeling of Mechanical Properties of Silica Fume-Based Green Concrete Using Machine Learning Techniques

**DOI:** 10.3390/polym14010030

**Published:** 2021-12-22

**Authors:** Afnan Nafees, Muhammad Nasir Amin, Kaffayatullah Khan, Kashif Nazir, Mujahid Ali, Muhammad Faisal Javed, Fahid Aslam, Muhammad Ali Musarat, Nikolai Ivanovich Vatin

**Affiliations:** 1 Department of Civil Engineering, COMSATS University Islamabad, Abbottabad Campus, Abbottabad 22060, Pakistan; 2 Department of Civil and Environmental Engineering, College of Engineering, King Faisal University (KFU), Al-Hofuf P.O. Box 380, Al Ahsa 31982, Saudi Arabia; mgadir@kfu.edu.sa (M.N.A.); kkhan@kfu.edu.sa (K.K.); 3 Department of Civil Engineering, School of Engineering, Nazabayev University, Astana 010000, Kazakhstan; kashif.nazir@nu.edu.kz; 4 Department of Civil and Environmental Engineering, Universiti Teknologi PETRONAS, Bandar Seri Iskandar 32610, Malaysia; mujahid_19001704@utp.edu.my (M.A.); muhammad_19000316@utp.edu.my (M.A.M.); 5 Department of Civil Engineering, College of Engineering in Al-Kharj, Prince Sattam Bin Abdulaziz University, Al-Kharj 11942, Saudi Arabia; f.aslam@psau.edu.sa; 6 Peter the Great St. Petersburg Polytechnic University, 195291 St. Petersburg, Russia; vatin@mail.ru

**Keywords:** green concrete, industrial waste, predictive modeling, machine learning, cross-validation, sensitivity analysis

## Abstract

Silica fume (SF) is a frequently used mineral admixture in producing sustainable concrete in the construction sector. Incorporating SF as a partial substitution of cement in concrete has obvious advantages, including reduced CO_2_ emission, cost-effective concrete, enhanced durability, and mechanical properties. Due to ever-increasing environmental concerns, the development of predictive machine learning (ML) models requires time. Therefore, the present study focuses on developing modeling techniques in predicting the compressive strength of silica fume concrete. The employed techniques include decision tree (DT) and support vector machine (SVM). An extensive and reliable database of 283 compressive strengths was established from the available literature information. The six most influential factors, i.e., cement, fine aggregate, coarse aggregate, water, superplasticizer, and silica fume, were considered as significant input parameters. The evaluation of models was performed by different statistical parameters, such as mean absolute error (*MAE*), root mean squared error (*RMSE*), root mean squared log error (*RMSLE*), and coefficient of determination (R^2^). Individual and ensemble models of DT and SVM showed satisfactory results with high prediction accuracy. Statistical analyses indicated that DT models bested SVM for predicting compressive strength. Ensemble modeling showed an enhancement of 11 percent and 1.5 percent for DT and SVM compressive strength models, respectively, as depicted by statistical parameters. Moreover, sensitivity analyses showed that cement and water are the governing parameters in developing compressive strength. A cross-validation technique was used to avoid overfitting issues and confirm the generalized modeling output. ML algorithms are used to predict SFC compressive strength to promote the use of green concrete.

## 1. Introduction

Greenhouse gas (GHG) emissions are considered the main cause of global warming, with CO_2_ as the most plentiful gas and having the greatest effect of all GHGs [1,2]. The cement industry accounts for around 5–7% of global CO_2_ emissions [3]. Concrete is a commonly used building material due to its mechanical and durability properties [4]. About 8% of CO_2_ is emitted due to the manufacturing process of concrete, which leads to global warming [5,6,7]. There is an estimated 20 billion tons of concrete produced annually, making it the second most widely used substance in the world after fresh-water. Aside from its benefits, concrete has a malignant effect on the Earth and human health and has adverse long-term effects on the natural environment and atmosphere [8]. It pushes the human footprint outwards by generating living space out of the air, spreading across rich topsoil, and causing biodiversity. The biodiversity crisis is a highlighted issue in research studies that it is one of the major threats to a sustainable environment and is primarily driven by urbanization. For hundreds of years, humanity has been eager for the advantages of concrete and not wanted to consider the environmental disadvantages. However, now the equilibrium will slide in the other direction. At the moment of disorienting transition, solidity is an enticing attribute that causes more challenges than something positive can fix [9].

During cement manufacturing, clinker production is the most energy-intensive process. During the formation of clinkers, almost half of the CO_2_ is emitted, and the rest of the CO_2_ is emitted during other cement manufacturing processes. This large emission of CO_2_ during clinker formation is due to the presence of calcareous and clay minerals in the kiln. For the production of a ton of cement, almost 900 kg of CO_2_ is re-leased. It must be heated to very high temperatures to form clinkers. Clinker is grounded to a fine powder and then mixed with gypsum to create cement. (Ca_3_SiO_5_) also known as alite, formed during clinker formation contributes too much of the initial strength. However, alite also must be kept at a temperature of 1500 °C during this process [10,11,12]. Some research suggests that alite can be replaced by other naturally available minerals with a roasting temperature less than alite. The reduction in carbon emissions from concrete has been a matter of concern both for the academic and industrial sectors [13]. Many approaches are suggested to address this issue. One of the possible solutions is the total or partial replacement of cement with some other mate-rial that may be readily available in nature [14,15,16]. Supplementary cementitious materials, such as silica fume (SF), have been used to partly substitute cement in concrete mixtures to minimize CO_2_ emissions from the cement industry [17,18,19,20].

SF is a major by-product of the silicon metal industry. Silicon metal is a semi-metallic element having several characteristics of metals. After oxygen, silica is the second most readily available element in the Earth’s crust in various forms of silicon dioxide or silicates but is readily available in its pure state [21,22].

**Figure 1:**
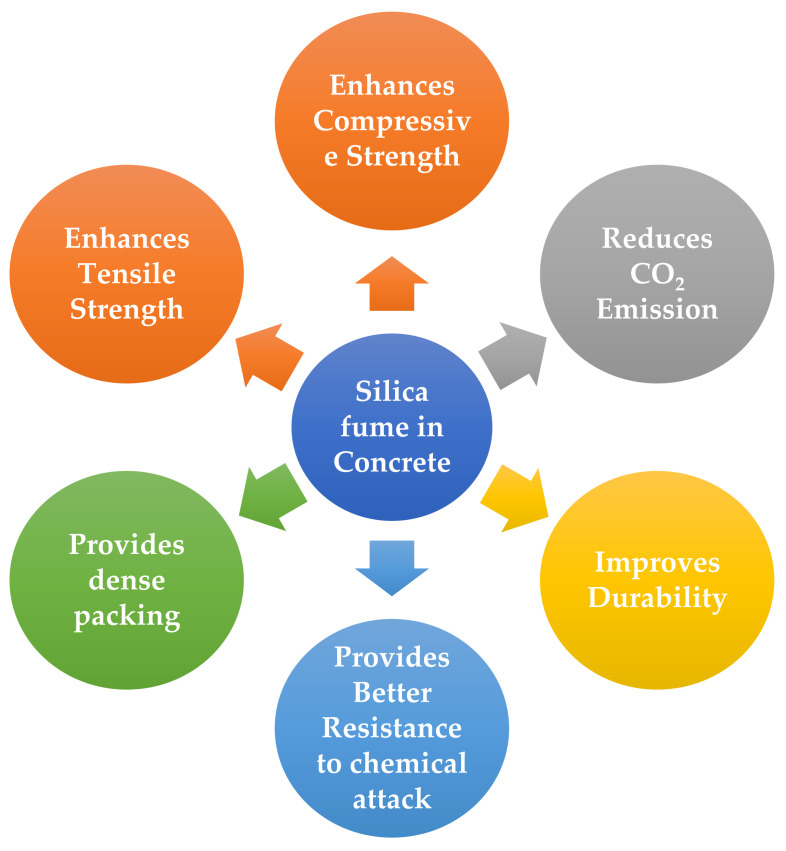
Silica fume benefits in concrete.

SF is a hazardous material and has malignant impacts on the atmosphere and its surroundings. Nearly all the silica fume was released into the atmosphere till the mid-1970 s. As the environmental concerns regarding SF developed, it was used in numerous applications. SF has very fine particles and contains a high amorphous silicon dioxide content, making it a highly pozzolanic material. It has a completely amorphous structure, due to which it is highly reactive. They are spherical and have a large surface area. SF particles are 100 times smaller than cement particles, so they are completely packed with the cement grains, and they also react with calcium hydroxide to form more CSH, which leads to the earlier strength [23,24,25]. It has dense packing due to its very small size, and hence it reduces the permeability. These properties of silica fume, when added to concrete, enhance the overall strength of concrete. Moreover, SF concrete has been widely used in high-strength and high-performance concrete for highway bridges, marine structures, and parking decks due to its utmost properties, as shown in Figure 1 [17,26].

Different experimental work has been performed to evaluate concrete’s short and long-term mechanical properties by replacing the different fine aggregate or cement levels with SF [27,28,29]. The literature suggests that the mechanical properties of SF, including compressive strength, initial strain due to creep, and modulus of elasticity, improved by replacing 15% of SF content. However, higher concentration causes a de-crease in concrete creep over the long term. The development of SF-based concrete strength depends on the curing temperature, material size, and silica content. The major contribution to strength under normal curing temperature takes place between 3 and 28 days. After 28 days, the additional strength due to SF is not appreciable. The re-placement of cement with SF between 5% and 25% with water to binder ratio in the range of 0.26–0.42 increases the compressive strength by about 6–30% [15,30]. The compressive strength of silica fume concrete (SFC) is significantly increased by varying water–cement ratios between 10% and 20% [31,32]. By increasing the water–cement ratio of SFC, a decrease in the concrete’s overall strength was observed. The compressive strength of concrete is decreased by 27% after 28 days by increasing the water–cement ratio by 0.05% with 15% SF content [31]. The properties of concrete are affected by many factors, including the mixed proportion of cement, sand, aggregate, and water. The mixing ratio of these materials determines the strength and durability of concrete. The anomalous behavior is observed for the mechanical properties of concrete at different mix ratios. A relationship between the mechanical properties of SF and the proportion of materials used in concrete is needed to promote sustainable development to cater to this behavior and promote the use of SF in concrete on a large scale. To achieve this, different modeling techniques from artificial intelligence are adopted, and empirical models are established to encourage sustainable development. Basic mechanical properties, including compressive strength and splitting tensile strength, must be taken into consideration for SFC design. SFC mixtures must also be optimized in terms of cost to achieve desirable properties by effectively proportioning SFC components. Traditionally, test lots are prepared in the laboratory to fulfill these criteria and meet construction specifications [32,33]. As only a limited number of tests can be produced in a laboratory, experimental methods can generate well, rather than best-performing proportions of SFC mixtures. The computational modeling approaches may be an alternative way of tackling the time-taking aspect of laboratory-based mixture optimization. These approaches firstly create the objective functions among the inputs (concrete constituents) and the outputs (properties) and use optimization algorithms to find the best concrete mixes. The objective functions are traditionally designed for linear or nonlinear models [34]. However, due to the strongly nonlinear relations between concrete properties and controlling variables, coefficients of these models cannot be precisely calculated [6]. Therefore, machine learning (ML) techniques are being used by researchers for modeling concrete properties.

In the past, various machine learning algorithms were used to predict concrete properties, such as modulus of elasticity, compressive strength, and splitting tensile strength. Amongst ML algorithms, multi-layer perceptron neural network (MLPNN) [35,36,37], support vector machine (SVM) [38,39], genetic engineering programming (GEP) [40,41,42,43], and deep learning (DL) [44,45,46], were mostly used. Ref. [47] employed SVM combined with K-Fold cross-validation, ANN, and Decision tree (DT) for predicting concrete strength degradation in the marine environment. It was concluded that SVM predicted the desired results with more accuracy and showed superior performance than the other two methods. Moreover, the SVM-based study was extended by [48] to a more complex screen and measured the unrestricted compression capacities of the cement–sand cockle-coated mixtures. Ref. [49] worked on an ANN approach to predict copper slag and nano-silica concrete strength. Similarly, efforts were made to predict the compressive and flexure strength of lightweight concrete with carbon fiber reinforcement [50]. The ANN technique provided better accuracy with R^2^ = 0.99 and 0.96 for compressive and flexural strength. Similarly, ref. [51] prophesized the compressive strength of recycled aggregate concrete and construction waste concrete using ANN. Likewise, ref. [52] employed ANN, DT, SVM, and linear regression methods to predict compressive strength. It was found that the DT method predicted the compressive strength results with the least error and showed superior performance compared to others. Ref. [53] developed models using GEP and ANN to predict the strength characteristics of geopolymer self-compacting concrete using raw materials. The author concluded that the GEP model outclassed the ANN model with the provision of expression for predicting output parameters by giving an empirical relationship. Similarly, ref. [54] studied the precedence of ANN in predicting the compressive strength of concrete. Mathematical expressions for formulating the said output were developed utilizing input parameters. In general, two methods of ML are used for modeling and predicting. Firstly, there is the traditional solution built on a single in-dependent paradigm, while secondly, there are collective learning algorithms, including boosting, bagging, and random forests created on many components of the data-base [55]. Individual ML models have weak learners who tend to produce overfitting of the data. Results show that these established approaches to ensemble learning are more exact than traditional single ML models [56]. First of all, training data are used to train weak learners in ensemble modeling. Weak learners are then incorporated into strong learners. Weak learners are trained based on individual learning methods, such as DT, SVM, and MLPNN. Consequently, the ensemble learning models provide accurate and robust predictions [57,58]. Ensemble ML techniques can effectively model multifaceted phenomena, such as SFC-containing waste materials. Most recent research has focused on improving the efficiency of ML modeling through the generation and use of ensemble-learning methods through classifiers [59]. Of course, the recent prediction modeling studies have shown that ensemble approaches are becoming more and more common as they usually produce more accurate results than individual base learners. Ref. [6] studied the efficacy of ensemble techniques in predicting the compressive strength of high-performance concrete using DT, MLPNN, SVM, and random forest (RF) techniques. Individual learners and ensemble learners for SVM, MLPNN, and DT with bagging and boosting were employed. It was concluded that ensemble techniques enhance the prediction accuracy of the models with superior performance. Similarly, ref. [60] compared different data-mining methods for the intensity forecast of environmentally friendly and renewable concrete according to their specific nature. Their study suggested that ensemble learning methods, when combined with individual regression and predictive modeling techniques, increase the efficiency of the models. Ref. [61] anticipated the compressive strength of concrete for 28, 51, and 90 days using DT, RF, and ANN. The correlation coefficient R^2^ and root mean square error (RMSE) was used as statistical indicators for the methods employed. Based on these statistical parameters, it was found that RF forecasted the best results followed by ANN. Ref. [62] also discussed the usefulness of ensemble learning techniques in accurately estimating the strength of reinforced concrete materials. Similarly, Table 1 summarizes the work performed by researchers using waste materials on machine learning.

The implication and originality of this research are twofold. Firstly, DT and SVM were applied to predict the compressive strength of SFC considering boosting with AdaBoost as an ensemble model for the prediction aspect. Secondly, ML techniques were then compared using statistical tools. According to the authors’ understanding, the literature lacks a similar study utilizing ensemble ML modeling for SFC. Various statistical indicators were used to check the performance of ML techniques for prediction accuracy. In this study, an attempt has been made to promote the use of SF in concrete, and studies have been conducted to reduce carbon footprints. The aim of this study is to make concrete greener by using computational techniques in utilizing SF as an additive or as a replacement in concrete for more sustainable development. This paper deals with modern ML techniques to study the behavior of SFC. Moreover, efforts are made to come up with the most eco-friendly concrete using these ML techniques.

## 2. Methods and Modeling

### 2.1. Overview of Artificial Intelligence (AI)

Structural engineering problems are influenced by several factors and are not repetitive. Before the enhancement in the artificial intelligence (AI) field, different classical models, including linear regression and nonlinear regression, were developed by engineers. These methods are not certain, and one cannot rely on them. Moreover, their accuracy was not much appreciable, and they were time-consuming. AI is the best alternative approach to classical modeling techniques. Moreover, AI-based systems are good substitutes for identifying engineering design criteria where experimentation is unavailable, leading to substantial human time and effort savings in experiments. AI can also speed up decision-making, reduce error rates, and improve computational efficiency [60].

### 2.2. Machine Learning Algorithms

**Figure 2:**
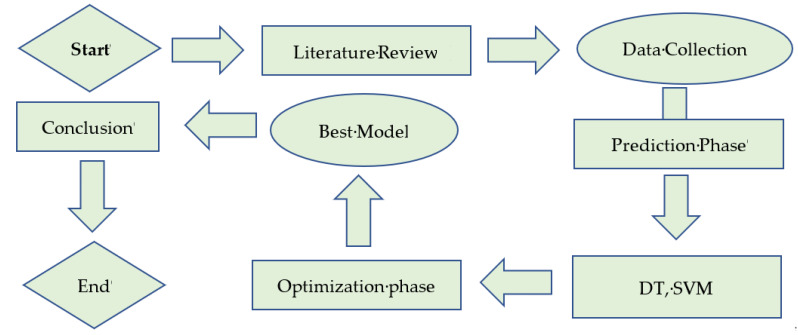
Machine Learning Algorithms.

Machine learning (ML) is one of the emerging technologies in the field of AI, which is frequently used in the construction industry to predict the behavior of mate-rials [6]. The current study employed to predict SFC’s compressive and split tensile strength by utilizing ML approaches, including DT and SVM, as illustrated in Figure 2. The said approaches are recommended by the researchers in predicting the mechanical properties of concrete. Furthermore, the modeling strength of concrete is predicted by using ensemble learners. The brief introduction to AI and ML approaches adopted in the present research are stated in the subsequent section.

ML models are very significant in terms of computational efficiency and processing time. They reduce the error rates to almost negligible compared to classical models. In this paper, an empirical model between the mechanical properties of SFC and mix proportions using the different ML techniques is established. Then the results are compared to predict the best model among these. This paper is concerned with DT and SVM among the major ML techniques. Modeling techniques used in this study are briefly discussed in the subsequent section.

#### 2.2.1. Decision Tree

DT is the predictive modeling technique used in AI for regression and classification problems. DT is based on a set of if-else statements and classifies according to the conditions. C4.5 is an international ML standard that demonstrates some efficiency. This program is a benchmark for the majority of DTs used in AI. C4.5 uses a heuristic entropy content measure to build the trees. This is because they can build incomprehensibly large trees with DT learners [48].

**Figure 3:**
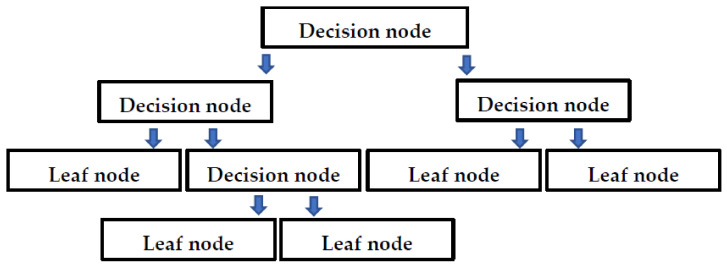
Flow chart of the Decision tree.

It consists of several nodes, also known as leaves, as illustrated in Figure 3. A test is applied at each leaf, which sends a query to the branches of that node. This loop will continue until the query arrives at the terminal leaf. The value returned as the contribution of the tree is correlated with each leaf node. This leaf node should focus on building the smallest tree by focusing on the major attributes first. An important at-tribute is organizing samples into groups. After the first attribute splits the samples, the remaining samples become DT problems themselves but with fewer samples and one less attribute. These subtrees with less but important attributes can overcome the complexity. The more samples at a node mean a higher complexity level. A homogenous node has a sample of one class, which reduces the complexity. The node aim is to grow trees by recursively trying to obtain leaf nodes that are as pure as possible by reducing the classes of the sample [49].

#### 2.2.2. Support Vector Machine (SVM)

**Figure 4:**
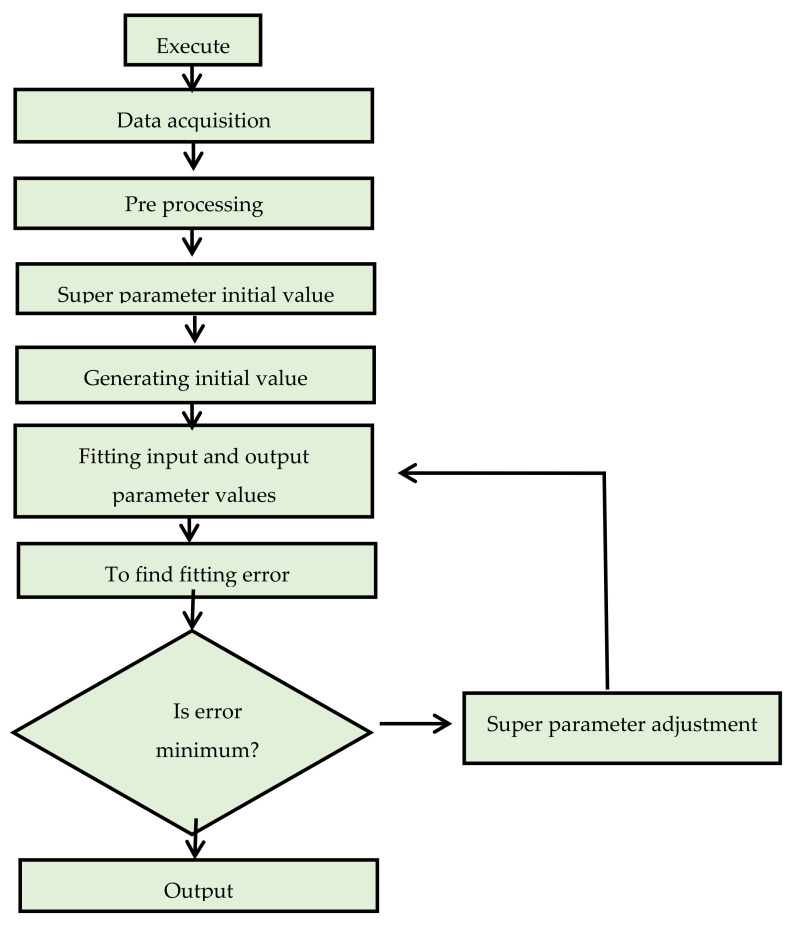
Flow chart of Support Vector Machine.

SVM is a supervised learning method provided by the dataset for input–output mapping. SVM models are used to solve classification and regression problems [54]. However, SVM is mainly employed in problems of classification. In this algorithm, x is a dimensional space where n is the number of features/inputs based on the model. The classification in SVM is performed by differentiating between two classes with the help of a hyperplane. Each data point is plotted as an x-dimensional space point (where n is the number of features) where the value of each feature is the value of a particular co-ordinate. After acquiring and detecting the number of input variables, an initial value is generated, and the output values are predicted. Using statistical parameters, these values are compared. Subsequently, classification by evaluating the hyper-plane is performed that distinguishes the two classes (input and output) very well [38]. The flow chart of SVM is presented in Figure 4.

### 2.3. Modeling Dataset and Model Development

**Figure 5:**
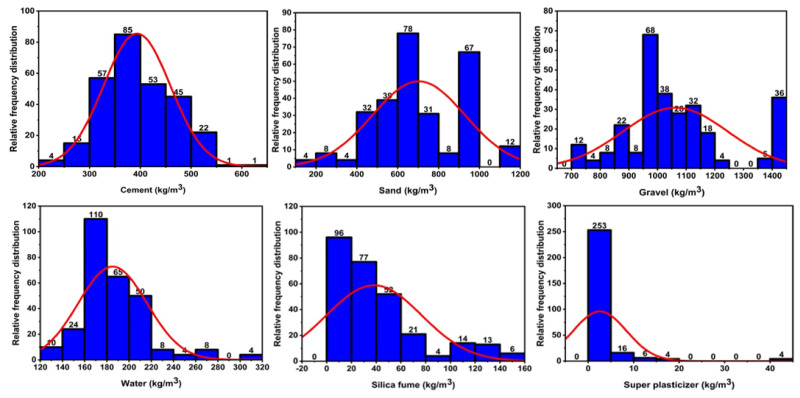
Relative frequency distribution of parameters to compressive strength; cement, sand, gravel, water, silica fume, and super plasticizer.

The silica fume concrete (SFC) database was built up from 22 internationally published studies available in the literature [6,63,64,65,66,67,68,69,70,71,72,73,74,75,76]. The frequency distribution and statistical description of the database contain 283 compressive tests (f’c), as shown in Figure 5. The mean, standard deviation, median skewness of metric, and maximum and minimum ranges of input parameters are listed in Table 2. It is suggested that the minimum ratio between the input variables and the database should be three, and for accurate models, it should be higher than five [77]. In this study, with the database of 283 for compressive strength with 6 input variables, the ratios are significantly higher, i.e., 47.17. Before developing a model, the input selection is the main process that affects the properties of the SFC. The most dominant constituent on the properties of concrete is sorted out to develop a generalized function. The properties of concrete are examined to be the function of Equation (1).

(1) $$fc,\;\left(MPa\right)=f\left(C,FA,CA,W,SF,SP\right)$$

where

*C* = Cement,

*FA* = Fine aggregate,

*CA* = Coarse aggregate,

*W* = Water,

*SF* = Silica fume,

*SP* = Superplasticizer.

These factors are the main constituents of SFC. Moreover, these factors influence the strength prediction of the model. The relation between these input variables is determined with the desired output (f’c). The minimum and maximum ranges of input variables that are the functions of outputs with their ranges are mentioned in Table 3. Other factors influence the properties of concrete, but their contribution to the desired output on SFC is negligible. The machine learning empirical models were trained in the training data (80% of the total data) and subsequently applied to the validation data (20% of the total data) that measures the precision and accuracy of the model [78]. The database collected from the literature contains information about the SF replacement percentage, water-to-binder ratios, specific gravity of fine aggregate and SF, fineness modulus of SF, and fractions of superplasticizer to maintain the workability. A training set is used in a database to construct a model, while the built-in model is validated by test data (or validation set) [6].

**Table 2:** Statistical description of data in the model for compressive strength (Kg/m^3^).

Parameters	Cement	Fine Aggregate	Coarse Aggregate	Water	Silica Fume	Superplasticizer
**Statistical Description**						
Mean	393.48	702.90	1062.41	185.15	38.25	2.56
Std error	3.92	13.44	10.88	1.84	2.27	0.35
Median	383.15	653.00	1040.00	175.00	26.25	0.00
variance	4359.48	51,138.84	33,530.89	963.29	1469.97	34.80
Std. dev	66.02	226.13	183.11	31.03	38.34	5.89
Kurtosis	−0.15	−0.51	0.20	3.66	0.57	30.00
Skewness	0.15	0.11	0.61	1.50	1.11	4.97
Range	376.00	985.36	728.00	178.87	150.00	43.00
Min	224.00	184.63	702.00	135.00	0.00	0.00
Max	600.00	1170.00	1430.00	313.87	150.00	43.00
Sum	111,354.90	198,941.50	300,663.20	52,397.59	10,827.33	726.11
Count	283	283	283	283	283	283
**Training Dataset**						
Mean	393.14	697.76	1067.67	185.80	36.78	2.65
Std error	4.41	14.67	11.94	2.15	2.56	0.42
Median	382.82	653.00	1040.00	176.00	26.25	0.00
variance	4404.11	48,659.21	32,197.86	1045.27	1483.09	40.60
Std. dev	66.36	220.59	179.44	32.33	38.51	6.37
Kurtosis	−0.14	−0.38	0.28	3.70	0.52	27.05
Skewness	0.13	0.11	0.65	1.57	1.11	4.83
Range	376.00	985.37	728.00	178.88	150.00	43.00
Min	224.00	184.63	702.00	135.00	0.00	0.00
Max	600.00	1170.00	1430.00	313.88	150.00	43.00
Sum	88,848.53	157,693.90	241,294.40	41,990.32	8313.19	599.42
Count	226	226	226	226	226	226
**Testing Dataset**						
Mean	394.85	723.64	1041.56	182.58	44.11	2.22
Std error	8.64	32.84	26.13	3.36	4.96	0.46
Median	390.00	653.00	990.00	175.00	29.62	0.00
variance	4255.64	61,470.40	38,931.08	642.77	1399.90	11.97
Std. dev	65.24	247.93	197.31	25.35	37.42	3.46
Kurtosis	−0.17	−0.93	0.05	0.46	1.07	9.21
Skewness	0.28	0.09	0.57	0.73	1.24	2.55
Range	302.00	932.82	728.00	125.70	150.00	19.00
Min	238.00	237.19	702.00	135.20	0.00	0.00
Max	540.00	1170.00	1430.00	260.90	150.00	19.00
Sum	22,506.35	41,247.52	59,368.84	10,407.28	2514.14	126.69
Count	57	57	57	57	57	57

### 2.4. Models Evaluation Criteria

The developed model performance on training or testing sets can be measured by computing statistical errors, such as mean absolute error (*MAE*), root mean square error (*RMSE*), root mean squared logarithmic error (*RMSLE*), and root square value (R^2^). However, the R^2^ value is also called the coefficient of determination and is considered the best among these for evaluating the model. With the advancement in the AI field, different modeling techniques have been adopted to develop prediction models for the mechanical properties of the resulting concrete. This study evaluates the models by statistical analysis by computing error metrics. These metrics can give different in-sights into the model’s errors.

Furthermore, the coefficient of variance and standard deviations are also used to evaluate the model’s performance. In this study, the model accuracy and validation are justified by its coefficient of determination. The R^2^ value for the model between 0.65 and 0.75 shows good results, while less than 0.50 depicts unsatisfactory results. The value of R^2^ can be calculated using Equation (2).

(2) $$R^{2}=\frac{\sum_{i=1}^{n}\left(M_{i\;}-{\bar{M}}_{i}\right)(P_{i\;}-{\bar{P}}_{i})}{\sqrt{\sum_{i=1}^{n}{\left(M_{i}-{\bar{M}}_{i}\right)}^{2}\sum_{i=1}^{n}{\left(P_{i\;}-{\bar{P}}_{i}\right)}^{2}}}$$

*MAE* is the average of absolute error when each input entity has the same weight. It corresponds to the difference between prediction and actual observation. The absolute value is taken to remove the negative sign. It determines the absolute size of the errors, and the units are identical to the output units. A model with an *MAE* value within a range can have punctually very high errors. It is calculated by using Equation (3).

(3) $$MAE=\frac{1}{n}\sum_{i=1}^{n}\left|P_{i}-M_{i}\right|$$

*RMSLE* considers the relative error between the predicted and the actual value. It is defined as the difference between the log of the anticipated value and the log of the actual value. Equation (4) is used to calculate *RMSLE*, where *x* is the predicted value and y is the actual value. This equation is helpful once it comes to right-skewed out-puts since the log transform renders the target spread more naturally.

(4) $$RMSLE=\sqrt{\frac{1}{N}\overset{N}{\underset{i=1}{\sum }}{\left(\log\left(x+1\right)-\log\left(y+1\right)\right)}^{2}}$$

*RMSE* is the square root of the average of squared variations between estimation and actual measurement. It measures the mean square magnitude of errors. It is the standard deviation of the predicted error. Large exceptions, such as outliers, are given more weight in this calculation, so large differences squared become greater, and small differences squared become smaller. The root means square error measures the aver-age prediction error made by the model in predicting the output for an input, where *P* is the predicted value and *M* is the measured value. The lower the *RMSE*, the better the model. An *RMSE* value ≥ 0.5 reflects the poor ability of the model to accurately predict the data. *RMSE* can be calculated by using Equation (5). Table 4 provides an overview of the range of different statistical parameters.

(5) $$RMSE=\sqrt{\frac{\sum_{i=1}^{n}{\left(P_{i}-M_{i}\right)}^{2}}{N}}$$

## 3. Results and Discussion

### 3.1. Formulation of Compressive Strength and Split Tensile Strength of SFC

Ensemble approaches are used to improve the degree to which data extraction and machine learning techniques (ML) are recognized or predicted. These methods also tend to decrease excessive training issues by integrating and combining various weaker analytical models (sub-models). By intelligently adjusting training data, the development of several sub-models/classification components (1, 2, …, m) will help a better learner. More precisely, a combination of qualified sub-models with averaging/voting methods can produce the optimum parametric/predictive model. Bagging is one of the more traditional bootstrap samplings and collating benefits modeling methods. The initial training range replaces component templates during the bagging phase. Certain data points could appear several times in product models while others do not. Then, combining the output of the variable models calculates the final output. Similar to the bagging strategy, the boosting method creates a cumulative model that results in several more accurate components than a model. In other words, the boosting uses weighted averages of dependent sub-models to place sub-models in the last model. This study covers base learners, such as DT and SVM, together with boosting, for forecasting the compressive of SFC. Parameter models used in the tuning techniques of the ensemble can (i) be linked to the optimal sample learner number and (ii) be associated with learning rates and other parameters that directly affect the ensemble method.

**Figure 6:**
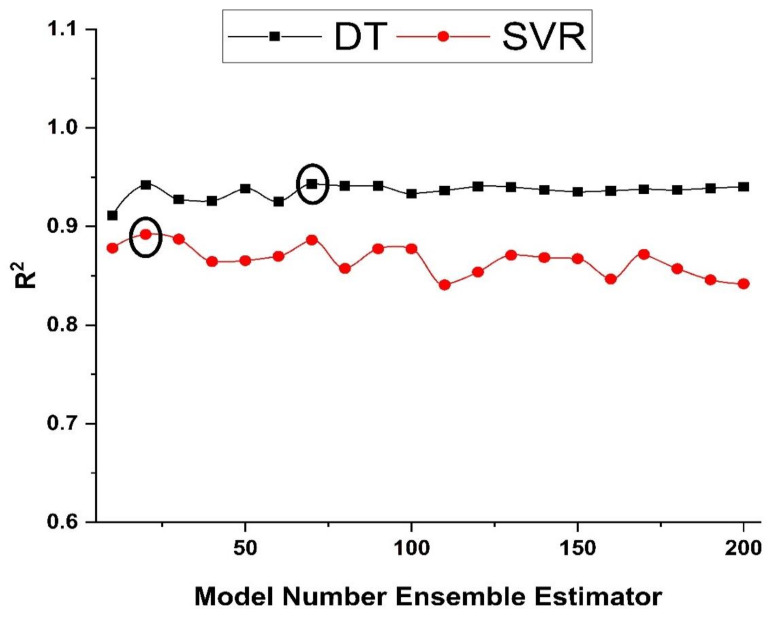
Coefficient of determination for ensemble models with the various number of component sub-models for compressive strength.

In this research, 20 boosting ensemble models of 10, 20, 30, …, 200 component sub-models were developed for each base learner for the choice of the optimal array of sub-models, and the best structures were chosen for training the data set for the respective ML system based on the high determination coefficient (R^2^) values. Figure 6 shows the relation among the R^2^ of ensemble models with varying numbers of component sub-models for compressive strength.

#### 3.1.1. Modeling Outcome of Decision Tree

**Figure 7:**
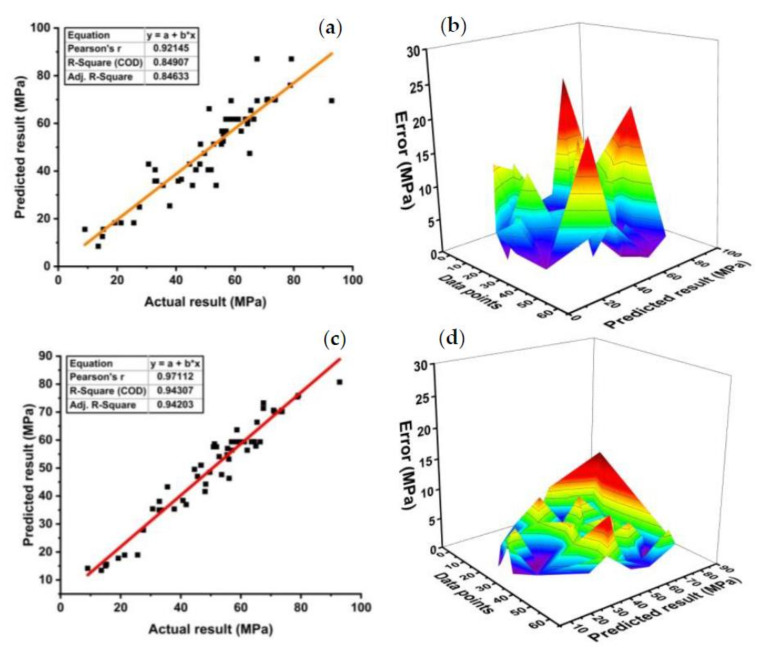
DT model results for compressive strength; (**a**) individual regression model, (**b**) error distribution between actual and target values of individual model, (**c**) ensemble regression model, (**d**) and error distribution between actual and target values of an ensemble model.

The prediction of compressive strength of SFC via DT gives superior performance against the actual results, as illustrated in Figure 7. The individual model gives accurate and good performance with R^2^ = 0.849, as depicted in Figure 7a. However, the ensemble model with boosting gives robust performance compared to the individual one, as depicted in Figure 7c. A comparison of the individual and ensemble methods for all the models is shown in Table 5. The robust performance of the ensemble model can also be correlated with its error distribution, as shown in Figure 7b,d. Figure 7b shows an average error of 5.46 MPa with the maximum and minimum errors of 23.33 and 0.0714 MPa, respectively, for the individual DT model. The average, maximum, and minimum error values are decreased using an ensemble algorithm to 3.57, 12.13, and 0.027 MPa, respectively, as shown in Figure 7d. These statistics depict that average, maximum, and minimum errors are improved by 34.62, 48.01, and 61.97 percent, respectively. Furthermore, 63.16 percent of the data of individual DT models indicate an inaccuracy of less than 5 MPa, whereas 17.54 and 12.28 percent of the data indicate errors between 5 and 10 MPa and 10–15 MPa, respectively. Moreover, 5.26 of the data shows the error between 15 and 20 MPa and 1.75 between 20 and 25 MPa. In contrast to the individual DT model, the data from the ensemble DT algorithm shows no inaccuracy over 15 MPa. Data from the ensemble DT model show that the error is 71.93 percent less than 5 MPa, whereas 26.32 and 1.75 percent of the data show that the error is between 5 and 10 and 10–15 MPa, respectively.

The ensemble DT model, when compared to the individual DT model, improves R^2^ by 11% for compressive strength. Relatively, ref. [6] showed an increase of 11 percent in boosting ensemble technique when compared with the individual DT model. Similarly, an enhancement of 12.2 percent was observed in predicting the compressive strength of fly-based concrete using the ensemble technique when compared with the individual DT model [58]. Accordingly, the values of DT metrics are satisfactory, and this algorithm can be utilized to accurately predict the model’s compressive strength. The accuracy of a model is highly dependent on the number of databases. This model consists of 283 data.

#### 3.1.2. Model Outcomes of Support Vector Machine (SVM)

**Figure 8:**
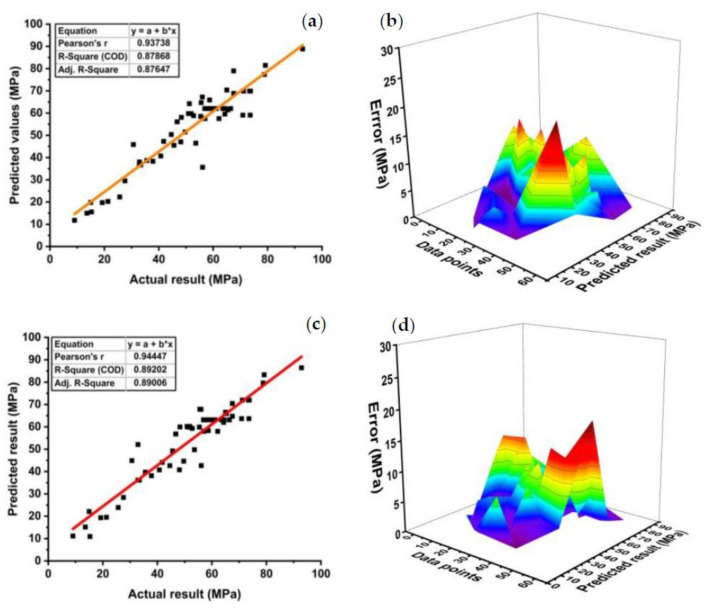
SVM model results for compressive strength; (**a**) individual regression model, (**b**) error distribution between actual and target values of individual model, (**c**) ensemble regression model, and (**d**) error distribution between actual and target values of an ensemble model.

Figure 8a shows the relation of predicted values with the target values with R^2^ = 0.87 for the SVM compressive strength individual model, while Figure 8c depicts R^2^ = 0.89 for the best ensemble SVM model. The red line in Figure 6 reflects the R^2^ in the SVM model developed to predict compressive strength with the model number estimator. Figure 8b depicts the maximum and minimum errors of 20.48 and 0.029 MPa, respectively, with an average error of 4.96 MPa for the individual SVM model. Moreover, the data indicate an error of 63.16 percent below 5 MPa, 24.56 percent between 5 and 10 MPa, 8.77 percent between 10 and 15 MPa, and 3.51 percent between 15 and 20 MPa. The error distribution of the SVM best compressive strength ensemble model, as seen in Figure 8d, depicts the maximum and minimum errors of 19.17 and 0.02 MPa, respectively, with an average error of 4.73 MPa. Average, maximum, and minimum errors were enhanced by 4.64, 6.4, and 31 percent, respectively. Moreover, the data show an error of 66.67 percent below 5 MPa, 21.05 percent between 5 and 10 MPa, 10.5 percent between 10 and 15 MPa, and only 1.75 percent between 15 and 20 MPa. The SVM boosting ensemble model enriches the R^2^ by 1.5 percent for compressive strength compared to the individual SVM model. Relatively, an enhancement of 8.43 percent was observed in predicting the compressive strength of high-performance concrete using the ensemble SVM model when compared to the individual SVM model [78]. However, an increase of only 0.21 percent was observed using the SVM ensemble model in predicting the deflection of reinforced beams when compared to the individual SVM model [79].

### 3.2. Comparison between Ensemble Models and GEP Model

**Figure 9:**
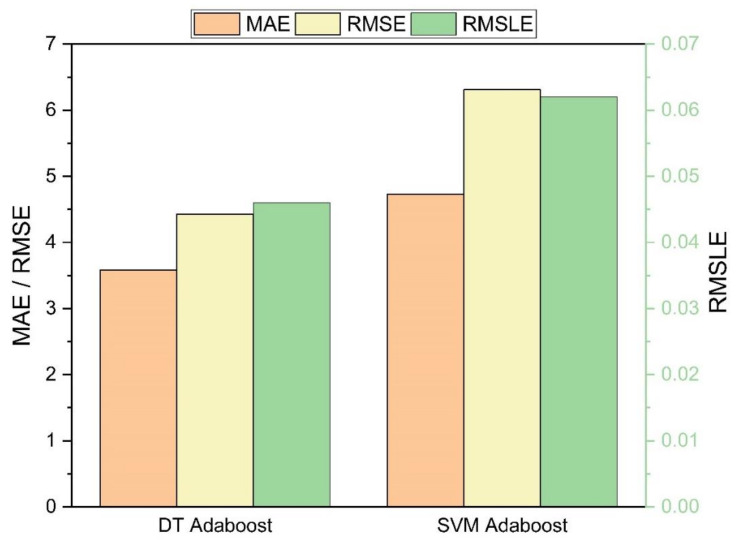
Comparison of errors for compressive strength.

To the author’s knowledge, no model has been developed to predict the mechanical properties of SFC. Consequently, this study has been employed to develop nonlinear regression models to predict the mechanical properties of SFC. Table 6 shows the statistical errors between the predicted and actual values. It can be observed from the statistical parameters that the actual and predicted values are closer for the DT model, which confirms the prediction accuracy of the DT model in forecasting the compressive strength of SFC. From Figure 9, it is deducted that the DT models show satisfactory results over SVM ensemble models with the same input variables for compressive strength of SFC.

### 3.3. Sensitivity Analysis

**Figure 10:**
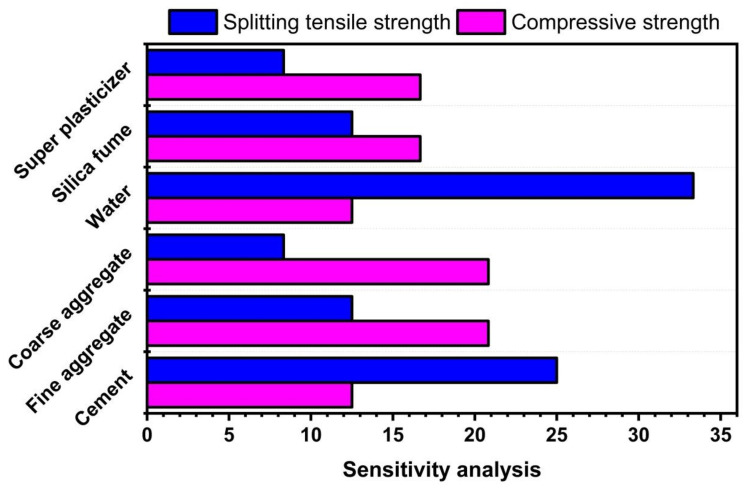
Contribution of input parameters to compressive and splitting tensile strength.

Six parameters, including cement, FA, CA, water, SF, and SP, were used as input parameters. Figure 10 shows the contribution of each input parameter in the development of the models. Water and cement have been shown to contribute more to compressive strength than FA, CA, and other additives. SF and SP played a modest role in developing both (DT and SVM) models.

### 3.4. Cross-Validation

Cross-validation is a statistical practice used to estimate the actual performance of the ML models. It is necessary to know the performance of the selected models. For this purpose, a validation technique is essential for determining the accuracy level of the model’s data. Shuffling the dataset randomly and splitting a dataset into k-groups is required for the k-fold validation test. In the described study, the data of experimental samples are equally divided into 10 subsets. It uses nine out of ten subsets, while the only subset is utilized to validate the model. The same approach of this process is then repeated 10 times for obtaining the average accuracy of these 10 repetitions. It is clarified widely that the 10-fold cross-validation method represents the conclusion and accuracy of the model performance well [58].

**Figure 11:**
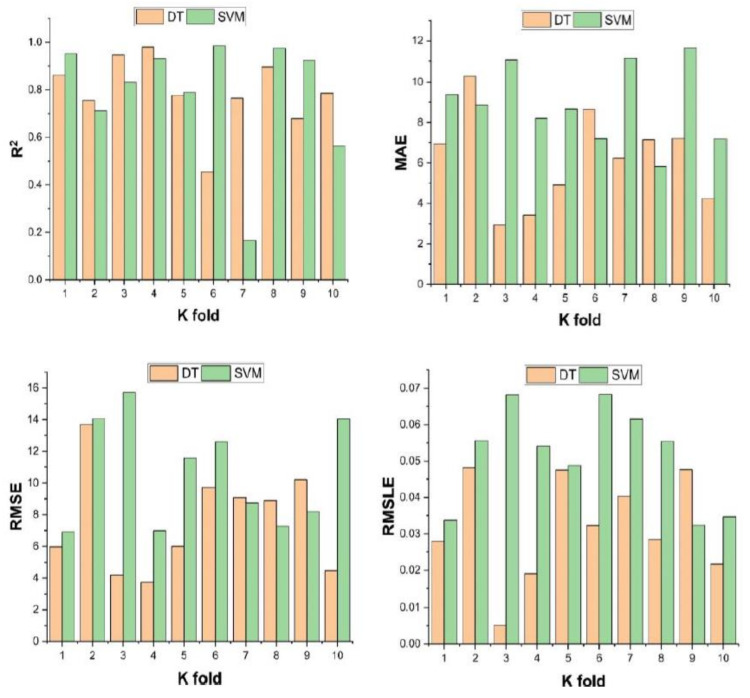
k-fold cross-validation for compressive strength.

Bias and a variance decrease for the test set can be checked by employing k-fold cross-validation. The results of the cross-validation are evaluated by a correlation co-efficient (R^2^), a mean absolute error (*MAE*), a mean square logarithmic error (*RMSLE*), and a root mean square error (*RMSE*), as illustrated in Figure 11. Both the models show fewer errors and better R^2^. The average R^2^ for DT model is 0.79 for compressive strength of ten folds with maximum and minimum values of 0.98 and 0.46, as shown in Figure 11. Similarly, the average R^2^ = 0.78 for SVM with a maximum and minimum value of 0.99 and 0.17, respectively, as shown in Figure 11. Each model shows fewer errors for validation. The validation indicator result shows that mean values of *MAE*, *RMSE*, and *RMSLE* come to be 6.20, 7.59, and 0.032, respectively, for the compressive strength DT model and 8.92, 10.61, and 0.051 for the compressive strength SVM model.

## 4. Conclusions

From the last two decades, soft computing techniques have been widely used for both linear and nonlinear systems of modeling to predict different properties of concrete. This study aimed to predict the compressive strength of SFC by using DT and SVM modeling. Compressive strength is the principal property of concrete, and there is no model that has been developed to predict the fc’ of SFC. After a detailed literature review, an extensive and reliable database was collected from the different research. The evaluation of models was performed by statistical parameters, including R^2^, *MAE*, *RMSE*, and *RMSLE*. The values of the statistical parameters indicated that both models could predict the compressive strength of concrete with reasonable accuracy. The ensemble model results are compared. For more verification, external validation and sensitivity analysis were also conducted. The R^2^ values of the best ensemble model for DT and SVM were obtained as 0.94 and 0.89, respectively.

The specific outcomes obtained from this study are,

- The results of this study indicated that ensemble models have higher accuracy for the prediction of data than individual models.
- After a detailed study, it was observed that among the ensemble models, the DT model showed the most accurate result for compressive strength compared to SVM, with prediction accuracy of 94% for DT and 89% for SVM.
- Different researchers have utilized silica fume in concrete in different percentages to enhance the mechanical properties of concrete. The accurate expressions and models can efficiently increase the utilization of hazardous SF in the concrete on the industrial level in construction practices rather than accumulating it as industrial waste. The replacement of silica fume with cement and determining its optimum percentage in concrete will help promote sustainable development by reducing energy consumption, landfilling, and greenhouse gas emissions.

## 5. Limitations and Directions for Future Work

An extensive and reliable database for compressive strength and split tensile strength was used. However, to provide a more general expression, including more input parameters and extending the database can provide the desired results. In addition, ML techniques can be combined with heuristic methods, including whale optimization algorithm, ant colony optimization, and particle swarm optimization, for better results. These methods can then be compared with the techniques employed in this study. Moreover, multi-expression programming (MEP) is an extended and improved form/version of GEP. GEP and MEP analysis should be employed and compared to overcome the limitations of ensemble algorithms.

## Figures and Tables

**Table 1:** Prediction of concrete properties by using waste material.

S.No	Algorithm Name	Notation	Dataset	Prediction Properties	Year	Waste Material Used	References
1	Artificial neural network	ANN	300	Compressive strength	2009	FA	[59]
2	Artificial neural network	ANN	80	Compressive strength	2011	FA	[60]
3	Artificial neural network	ANN	169	Compressive strength	2016	FA GGBFS SF RHA	[61]
4	Artificial neural network	ANN	69	Compressive strength	2017	FA	[34]
5	Artificial neural network	ANN	114	Compressive strength	2017	FA	[62]
6	Adaptive neuro fuzzy inference system	ANFIS	55	Compressive strength	2018	-	[63]
7	Random Kitchen Sink Algorithm	RKSA	40	V-funnel test J-ring test Slump test Compressive strength	2018	FA	[64]
8	Multivariate adaptive regression spline	M5 MARS	114	Compressive strength Slump test L-box test V-funnel test	2018	FA	[65]
9	Artificial neural network	ANN	205	Compressive strength	2019	FA GGBFS SF RHA	[66]
10	Random forest	RF	131	Compressive strength	2019	FA GGBFS SF	[67]
11	Intelligent rule-based enhanced multiclass support vector machine and fuzzy rules	IREMSVM-FR with RSM	114	Compressive strength	2019	FA	[68]
12	Support vector machine	SVM	-	Compressive strength	2020	FA	[69]
13	Multivariate	MV	21	Compressive strength	2020	Crumb rubber with SF	[70]
14	Biogeographical-based programming	BBP	413	Elastic modulus		SF FA SLAG	[71]
15	Support vector machine	SVM	115	Slump test L-box test V-funnel test Compressive strength	2020	FA	[72]
16	Adaptive neuro fuzzy inference system	ANFIS with ANN	7	Compressive strength	2020	POFA	[73]
17	Data Envelopment Analysis	DEA	114	Compressive strength Slump test L-box test V-funnel test	2021	FA	[74]

**Table 3:** The maximum and minimum range of silica fume concrete data for compressive strength.

Parameters	Abbreviation	Minimum	Maximum
**Input Variables**			
Binder	C	224	600
Fine aggregate	FA	184.6	1170
Coarse aggregate	CA	702	1430
Water	W	135	313.9
Silica Fume	SF	0	150
Superplasticizer	SP	0	43
**Output Variable**			
Compressive strength	fc’	5.66	95.9

**Table 4:** Range of errors for statistical parameters.

Assessment Criteria	Range	Accurate Model
*MAE*	(0, ∞)	the smaller the better
*RMSE*	(0, ∞)	the smaller the better
*MSLE*	(0, ∞)	the smaller the better
R^2^ value	(0,1)	the bigger the better

**Table 5:** R^2^ values of individual and ensemble models.

Approaches Employed	Output Parameter	Machine Learning Methods	Ensemble Models	Optimum Estimator	R Value
Individual algorithms	Compressive Strength	Decision tree	-	-	0.85
		Support vector machine	-	-	0.88
Ensemble boosting	Compressive strength	Decision tree—Adaboost	(10, 20, 30, …, 200)	70	0.94
		Support vector machine—Adaboost	(10, 20, 30, …, 200)	20	0.89

**Table 6:** Statistical errors for the models.

Models	MAE	RMSE	RMSLE	R^2^ Value
**Decision Tree Values**				
**Compressive strength**	3.58	4.43	0.046	0.94
**SVM**				
**Compressive strength**	4.73	6.31	0.062	0.89

## Data Availability

The data used in this research was collected from published literature.
